# Genetic Structure of the Polymorphic *Metrosideros* (Myrtaceae) Complex in the Hawaiian Islands Using Nuclear Microsatellite Data

**DOI:** 10.1371/journal.pone.0004698

**Published:** 2009-03-04

**Authors:** Danica T. Harbaugh, Warren L. Wagner, Diana M. Percy, Helen F. James, Robert C. Fleischer

**Affiliations:** 1 National Museum of Natural History, Smithsonian Institution, Department of Botany, Washington, D. C., United States of America; 2 Center for Plant Research and Department of Botany, University of British Columbia, Vancouver, British Columbia, Canada; 3 National Museum of Natural History, Smithsonian Institution, Department of Vertebrate Zoology, Washington, D. C., United States of America; 4 National Zoological Park, Smithsonian Institution, Washington, D. C., United States of America; American Museum of Natural History, United States of America

## Abstract

**Background:**

Five species of *Metrosideros* (Myrtaceae) are recognized in the Hawaiian Islands, including the widespread *M. polymorpha*, and are characterized by a multitude of distinctive, yet overlapping, habit, ecological, and morphological forms. It remains unclear, despite several previous studies, whether the morphological variation within Hawaiian *Metrosideros* is due to hybridization, genetic polymorphism, phenotypic plasticity, or some combination of these processes. The Hawaiian *Metrosideros* complex has become a model system to study ecology and evolution; however this is the first study to use microsatellite data for addressing inter-island patterns of variation from across the Hawaiian Islands.

**Methodology/Principal Findings:**

Ten nuclear microsatellite loci were genotyped from 143 individuals of *Metrosideros*. We took advantage of the bi-parental inheritance and rapid mutation rate of these data to examine the validity of the current taxonomy and to investigate whether *Metrosideros* plants from the same island are more genetically similar than plants that are morphologically similar. The Bayesian algorithm of the program structure was used to define genetic groups within Hawaiian *Metrosideros* and the closely related taxon *M. collina* from the Marquesas and Austral Islands. Several standard and nested AMOVAs were conducted to test whether the genetic diversity is structured geographically or taxonomically.

**Conclusions/Significance:**

The results suggest that Hawaiian *Metrosideros* have dynamic gene flow, with genetic and morphological diversity structured not simply by geography or taxonomy, but as a result of parallel evolution on islands following rampant island-island dispersal, in addition to ancient chloroplast capture. Results also suggest that the current taxonomy requires major revisions in order to reflect the genetic structure revealed in the microsatellite data.

## Introduction

Disentangling the interacting roles of genetics and the environment in morphological patterns across the tree of life is one of the main goals of systematics and population genetics. The characterization of genetic diversity and relationships between closely related plant taxa is often difficult due in part to a lack of sufficiently variable genes [1] that do not reflect the often striking morphological changes that many plant groups undergo in response to environmental fluctuations, or minor genetic changes [2]. For example in adaptive radiations, as often seen in island archipelagos, dramatic morphological changes can occur rapidly resulting in a diversity of taxa, with little, or no detectable genetic differences [3], [4].

Determining the relationships and patterns of genetic diversity within the polymorphic Hawaiian *Metrosideros* (Myrtaceae) complex, known locally as *‘ohi‘a* or *‘ohi‘a lehua*, has been particularly challenging. The most recent comprehensive taxonomic treatment of *Metrosideros* recognizes about 50 species in the genus, which range across the Pacific from the Philippine Islands, south to New Zealand, and east to the Hawaiian Islands [5]. Five species are recognized in the Hawaiian Islands, including the widespread and variable species *Metrosideros polymorpha*, which is dominant in many different ecosystems on all of the major Hawaiian Islands, from sea-level to over 2,600 m in elevation. As the scientific name suggests, *M. polymorpha*, which is subdivided into eight varieties, is characterized by a multitude of distinctive, yet overlapping habit, ecological, and morphological forms, ranging from scandent shrubs to towering trees, from montane bogs to recent lava flows, and from large glabrous leaves to minute hairy ones [5].

The taxonomy of the group has long been problematic, as many of the morphological characters are continuous, often with multiple phenotypes present in a single population; many *M. polymorpha* individuals exhibit combinations of morphological traits that characterize two or three varieties, and individual plants may be intermediate between recognized taxa. To complicate the situation further, *Metrosideros* is heteroblastic (i.e. with developmental differences in leaf characteristics between juvenile and adult stages) [6], and epicormic shoots on adult plants have leaves with juvenile morphologies [7].

Because of the complex morphological variation in Hawaiian *Metrosideros*, they have become a model system to study ecology and evolution. A number of previous studies have investigated whether the dramatic morphological variation within *M. polymorpha*, and among other Hawaiian taxa, may be due to hybridization [7], genetic polymorphism, or phenotypic plasticity [8]. Although limited in sampling to a few populations within an island, early studies were able to conclude that morphological characters in *M. polymorpha* had strong environmental responses to water, nutrients, and temperature [6], [9], and that the leaf morphology of *M. polymorpha* varied as a function of elevation, substrate age, and annual precipitation [10]. In complementary common-garden and field studies, *M. polymorpha* morphology was determined to be partly environmentally and partly genetically controlled [8].

Our understanding of the morphological patterns in Hawaiian *Metrosideros* has increased with the advent of genetic methods. Several allozyme studies on populations within the islands of Maui and Hawai'i demonstrated that genetic diversity of *Metrosideros polymorpha* does not mirror that of the great morphological diversity present in these locations [11]–[13]. Another study of *M. polymorpha* based on RAPDs (random amplification of polymorphic DNA) and morphology showed that within the island of Hawai'i, a plant's locality was more predictive of genetic similarity than its morphology [14]. A Pacific-wide phylogenetic analysis of *Metrosideros* based on nuclear ribosomal ITS and ETS sequence data began to shed light on the relationships of Hawaiian *Metrosideros* to other Pacific taxa and indicated that the Hawaiian group likely originated from the Marquesas Islands [15]; they found a closer relationship between *M. collina* from the Marquesas Islands and the Hawaiian taxa than between *M. collina* in the Marquesas Islands and *M. collina* on other Pacific Islands. However, the data were insufficiently variable to resolve the relationships between Hawaiian taxa or between the Hawaiian taxa and the individuals sampled from the Marquesas Islands.

A recent study using chloroplast sequence data and morphology has provided a more in-depth analysis of the genetic and morphological patterns of *Metrosideros* across the Hawaiian Islands [16]. This study uncovered a strong geographical structure, with most individuals on each island more closely related to others on the same island, despite their morphologies. The study also found a strong biogeographic pattern where ancient dispersal events likely proceeded down the island chain from the older to younger islands; the Hawaiian Islands are an interesting area to study phylogeographic patterns because the islands are chronologically ordered, with Kaua'i the oldest (5.1 million years), and Hawai'i the youngest (0.5 million years to still forming). Several other plant and animal groups have demonstrated divergence patterns among species that are consistent with colonization from older to younger islands, often referred to as the “progression rule” [17], but this was the first study to find such a pattern within a species. However, because this previous study only included chloroplast data, they could not resolve whether hybridization and chloroplast capture accounted for some of the similar genotypes found in plants with different morphologies within islands [16]. Consequently, we sought additional evidence from nuclear DNA data, using the same set of samples (with additions) employed in the chloroplast phylogeny study [16].

The inconclusiveness and/or limited sampling of the above previous studies, plus the unisexual inheritance of CpDNA and the low variability of other nuclear markers attempted, emphasized the need for a study of *Metrosideros* using microsatellites to examine the genetic structure and gene flow of all *Metrosideros* species from across the Hawaiian Islands. This study represents the most complete taxonomic and geographic sampling of any study on Hawaiian *Metrosideros* to date. The bi-parental inheritance and rapid mutation rate [18] of microsatellite markers, regions of tandemly repeated basepairs, usually from 2 to 6 base-pairs in length [19], make them particularly useful in uncovering the genetic structure of closely related taxa and populations and are expected to provide better resolution than the chloroplast sequence data [16], which is the only other data available for a similarly comparatively wide sampling. Lastly, they are easy to score, making multilocus assessments of genetic structure across a wide sampling of individuals possible [20]. We genotyped ten nuclear microsatellite loci using primers developed for *M. polymorpha* from the island of Hawai'i [21]. Over 140 individuals were genotyped, representing all the species from across five of the major Hawaiian Islands. These data were used to investigate whether plants from the same locality are more genetically similar than plants with similar morphologies, and to examine the validity of the current taxonomy of Hawaiian *Metrosideros*.

## Methods

### Taxonomic sampling and DNA extraction

A total of 143 individuals were genotyped in this study - see Appendix S1 in the online Supporting Information for taxonomic, geographic, and herbarium voucher information. This sampling encompassed representatives from all the Hawaiian *Metrosideros* species and from all of the major Hawaiian Islands except Lana'i. The only Hawaiian taxon not represented is one of the eight varieties of *M. polymorpha* (var. *newellii*). Also included in this study were 11 samples of *M. collina* from Southern Polynesia (six samples from the Austral Islands and five from the Marquesas Islands); these were included because they were previously shown to be most closely related to Hawaiian *Metrosideros* [16]. Samples for this study were contributed by several collectors, with in some cases only a single or few individuals collected per population. Samples were selected to represent a wide taxonomic sampling from each island rather than large population samples per taxon. A majority (60.8%) of the individuals included in this study were previously analyzed for the chloroplast phylogenetic study [16] allowing for a direct comparison of the resulting patterns; the morphological character data analyzed in this study were also previously published [16]. For all samples, total DNA was extracted from silica-dried leaf material using a Qiagen DNeasy Plant Mini Kit. DNA was diluted from 1:10 to 1:50, based on the quality and age of the leaf material (with higher quality and younger material diluted more) before it was used for amplification.

### Microsatellite amplification and scoring

Ten polymorphic microsatellite loci (MePo501, MePo503, MePo505, MePo507, MePo508, MePo510, MePo511, MePo512, MePo513, and MePo514) were amplified using previously published primers that were developed for *M. polymorpha* from the island of Hawai'i [21]. Amplifications were performed in a 10 µL volume [1 µL 10× buffer (Applied Biosystems), 1 µL 10 µm dNTPs, 0.6 µL MgCl_2_, 0.1 µL *Taq* DNA polymerase (Applied Biosystems), 0.5 µL of each primer (forward and reverse), and 1 µL DNA template] using a PT-100 thermal cycler (MJ Research) under published conditions, except with annealing temperatures 1–2°C below published temperatures and annealing times increased to 30 seconds for all cycles [21]. Forward primers were fluorescently labeled on their 5′ end with either Hex, Fam (Sigma-Aldrich), or Ned (Applied Biosystems) dyes. Two to five dye-labeled PCR products were mixed so that multiple loci could be multi-loaded and analyzed in an ABI capillary sequencing machine (Applied Biosystems) using Rox standard (Applied Biosystems). Electropherograms were analyzed using genescan version 2.1 software (Applied Biosystems) and allele sizes were scored using genotyper version 2.5 software (Applied Biosystems).

### Data analyses

#### Definition of genetic groups

We used the Bayesian algorithm as implemented by the computer program structure version 2.2 [22] to define genetic groups within Hawaiian *Metrosideros* and determine the similarity of *M. collina* from the Marquesas Islands and the Austral Islands to Hawaiian taxa. structure analyses have been used to elucidate the genetic structure within a variety of taxa, from plants [23] and mosquitoes [24], to humans [25]. This algorithm infers genetic discontinuities from individual multilocus genotypes without any *a priori* knowledge of geographic location or taxonomy. The default settings of the program were used, including an admixture model. To determine the most likely number of groups (K) in the data, a series of analyses were performed from K = 1 through 20, using 40,000 burn-in and 100,000 repetitions, with ten iterations per K. These results were examined using the ΔK method [26] to identify the most likely number of groups in the data, which in this study was determined to be K = 13.

#### Analyses of population differentiation

To investigate the genetic structure further, and to test how the genetic diversity might be structured, several analyses of molecular variance (AMOVA) were conducted [27] using arlequin version 3.11 software [28]. For each analysis, the standard AMOVA settings were used, with 0.1 missing data level per site, and 10,000 permutations. Three loci (MePo503, MePo507 and MePo514) were excluded from most of the analyses because of too many missing data. Standard AMOVAs were used to test for the significance of grouping the data by island, species, and taxonomic variety. Lastly, several nested AMOVAs were conducted in arlequin with the same settings and number of permutations as above. Nested AMOVAs were conducted to test several groups, including species or varieties in islands and islands in species or varieties. For all AMOVAs using “variety” as a group, all individuals that had not been identified to variety, or were considered intermediate in form, were removed from those analyses.

## Results

### Characterization of microsatellite loci

The 10 polymorphic microsatellite loci used in this study are characterized in Table 1. For the 143 individuals genotyped, all but one locus (MePo514) could be genotyped across most of the individuals sampled, and two other loci (MePo503 and MePo507) were missing too many data to be included in the AMOVA analyses. The total number of alleles ranged from as few as 7 (MePo513) to as many as 51 (MePo503). The most extreme size range was from 179 to 393 base pairs (MePo503). The size ranges for all loci genotyped in this study were larger than those reported in the original publication [21] as this study included a wider sampling of taxa and localities.

**Table 1:** Characterization of *Metrosideros* nuclear microsatellite loci.

Locus	*N*	*k*	Size (bp)
MePo501	142	22	104–160
MePo503	117	51	179–393
MePo505	134	25	230–304
MePo507	126	20	220–280
MePo508	130	8	182–228
MePo510	137	23	216–294
MePo511	131	8	136–176
MePo512	132	38	138–308
MePo513	142	7	152–212
MePo514	66	17	166–212

*N* is the number of individuals genotyped in this study; *k* is the total number of alleles; and size is the range of base pair lengths of each locus.

### Definition of genetic groups

**Figure 1:**
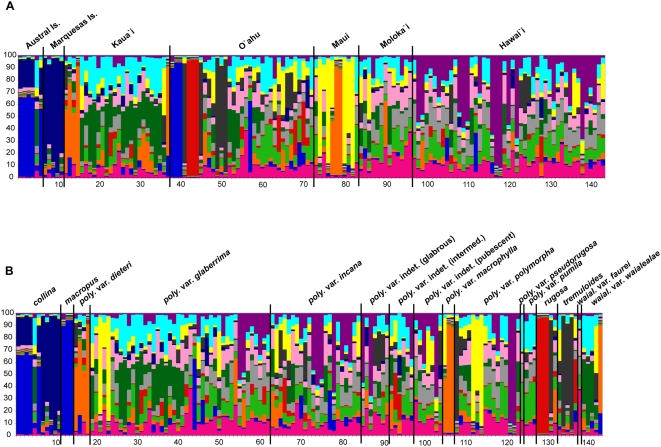
Genetic structure of *Metrosideros* using nuclear microsatellite data. Results from the Bayesian algorithm as implemented in the program structure. Each of the 143 individuals included in the analysis is represented by a single vertical bar, partitioned into 13 colored segments that represent the individual's probability of belonging to one of 13 (K = 13) genetic clusters. (*A*) Individual samples are grouped by island archipelagos (Austral Islands, Marquesas Islands), or by individual Hawaiian Island. The X-axis numbers follow Sample Numbers in Appendix S1. (*B*) Individual samples are grouped by *Metrosideros* taxon. The X-axis numbers follow Sample Numbers in Appendix S1.

The results of the Bayesian structure analysis identifying genetic groupings in the *Metrosideros* microsatellite data are illustrated in Figure 1. In Figure 1*A*, which is organized by island, five individuals from Tubuai in the Austral Islands group together (blue) and similarly to several individuals from O'ahu, while one individual from Rurutu in the Austral Islands has mixed group membership. All five individuals from the Marquesas Islands group together (dark blue), separately from those from the Austral Islands. All of the Hawaiian Islands are separated into a number of different genetic groups, represented by a diversity of colors in Figure 1*A*. A majority of specimens from Kaua'i are predominantly in the dark green group, which is scattered across most of the other Hawaiian Islands. However, five individuals from Kaua'i are placed in the orange group, which is also found in Maui. O'ahu is separated into a number of different groups, with no one group dominating the island; several individuals form a distinctive blue group, most similar to individuals from Tubuai (Austral Islands). In O'ahu, three individuals also form a very distinctive red group, not shared on any other island, while several other specimens form a dark grey group. Maui is predominated by a distinctive yellow group, with several individuals in the orange group, shared with a few individuals from Kaua'i. The group membership in Moloka'i is very heterogeneous, with no single group predominating on the island. Hawai'i is also heterogeneous, however, the purple group which is shared by many of the individuals in Hawai'i is not found throughout the other Hawaiian Islands.

Figure 1*B* illustrates the results from the structure analysis by separating the data along taxonomic lines. Interestingly, *M. collina* is separated into two groups, blue and dark blue, with one individual matching many of the individuals from Kauai; this blue group is also shared by all of the individuals of *M. macropus*. Most taxa are very heterogeneous, having individuals belonging to a number of different genetic groups, including *M. polymorpha* vars. *glaberrima*, *incana*, *polymorpha*, *pseudorugosa*, and *pumila* and *M. waialealae* vars. *faurei* and *waialealae*. However, two taxa do appear to form distinctive genetic groups, *M. rugosa* (red) and *M. tremuloides* (mostly grey), although not all individuals identified as *M. rugosa* or *M. tremuloides* were included in those genetic groups. *Metrosideros polymorpha* vars. *dieteri* and *macrophylla*, which occur on different islands, also group together (orange).

### Analyses of population differentiation

Results of the AMOVAs testing for genetic subdivision were mixed, revealing in the standard AMOVAs that the highest Fst value was for grouping the microsatellite data by species (Fst = 0.122), followed closely by taxonomic variety (Fst = 0.106), and then by islands (Fst = 0.090) (Table 2). However, results of the nested AMOVAs (Table 3) revealed that more of the variation is explained by islands than by either species or taxonomic variety.

**Table 2:** Analysis of molecular variance (AMOVA) results.

Main Category	Subdivisions	Variation	Df	Sum of Squares	Variance Component	Percentage of Variance	Fst
islands	Australs Islands, Marquesas Islands, Kaua'i, O'ahu, Maui, Molokai, Hawai'i	among popswithin pops	6279	60.050594.960	0.2122.133	9.0390.97	0.090
species	*M. collina*, *M. macropus*, *M. polymorpha*, *M. rugosa*, *M. tremuloides*	among popswithin pops	5282	43.601615.555	0.3042.183	12.2387.77	0.122
variety	*M. collina*, *M. macropus*, *M. polymorpha vars. dieteri*, *glaberrima*, *incana*, *macrophylla*, *polymorpha*, *pseudorugosa*, *pumila*, *M. rugosa*, *M. tremuloides*, *M. waialealae*	among popswithin pops	11240	68.132463.431	0.2301.931	10.6489.36	0.106

Genetic subdivision of the *Metrosideros* microsatellite by island, species, and taxonomic variety.

**Table 3:** Nested analysis of molecular variance (AMOVA) results.

Major Group	Minor Group	Variation	Df	Sum of Squares	Variance Components	Percentage of Variance	Fst/Fsc/Fct
islands	species	among groups	6	54.633	0.198	8.49	0.104
		among pops within groups	4	10.048	0.044	1.90	0.021
		within pops	211	436.241	2.0675	89.61	0.085
species	islands	among groups	4	23.741	0.103	4.32	0.123
		among pops within groups	6	40.940	0.190	7.99	0.084
		within pops	211	440.404	2.087	87.69	0.043
islands	variety	among groups	6	53.636	0.182	7.77	0.123
		among pops within groups	16	47.463	0.107	4.56	0.049
		within pops	205	420.323	2.050	87.67	0.078
variety	islands	among groups	11	53.156	0.064	2.76	0.118
		among pops within groups	11	47.940	0.209	9.01	0.092
		within pops	205	420.323	2.050	88.23	0.028

Genetic subdivision of the *Metrosideros* microsatellite data using several different major and minor groupings, including islands, species, and variety.

Even with the inclusion of *M. collina* from the Austral and Marquesas Islands, Fst values in this study are particularly low (islands = 0.09; species = 0.12) when compared to similar microsatellite studies in woody insular plants; for example the Fst of *Santalum insulare* between islands in French Polynesia is 0.50 [29], *S. austocaledonicum* between islands in New Caledonia is 0.35 [30], and *Magnolia sieboldii* ssp. *japonica* between populations in Japan is 0.49 [31]. However, Fst values in this study lie more in the range of other mainland woody species such as populations of *Vouacapoua americana* in French Guiana which has an Fst of 0.08 [32] and *Grevillea macleayana* in Australia with an Fst of 0.22 [33], although Fst values between studies are not directly comparable given different heterozygosity values.

## Discussion

The main objective of this study, based in part on some recent provocative chloroplast DNA analyses [16], was to investigate whether the genetic diversity of Hawaiian *Metrosideros* is geographically or taxonomically structured. The structure results using nuclear microsatellite data presented here reveal a much more complex assignment of individuals to groups than by only island or taxonomy (Figure 1), indicating that the evolution and current gene flow may be much more complicated, with several competing forces leading to the morphological and genetic patterns we see today. The results from the microsatellite analyses provide support for several of the findings based on the chloroplast sequence data [16], most notably some evidence for genetic differentiation by islands as determined by the nested AMOVA results, likely due to the differentiated genetic groups in the Austral Islands (blue), Marquesas Islands (dark blue), Maui (yellow), and Hawai'i (purple) (Figure *1A*). However, a marked difference between these studies was that the microsatellite data grouped *M. collina* from the Austral Islands (Tubuai) with *M. macropus* from O'ahu, while in the nuclear and chloroplast DNA studies [15], [16], the Marquesas Islands shared more genetic similarity with individuals from the Hawaiian Islands than to those from the Austral Islands. Although not likely, due to the inclusion of ten independent loci, this may have resulted from size homoplasy; due to the rapid mutation rates of microsatellite regions, electromorphs of the same size may not be the result of common descent [34]–[36]. Another difference in the two studies is that that all individuals of *M. polymorpha* var. *dieteri* formed a group in the microsatellite analysis, but were resolved as polyphyletic in the chloroplast phylogeny. Lastly, most individuals of *M. tremuloides* and *M. rugosa* formed distinctive groups in this study, but chloroplast sequence data [16] may not have had sufficient variability to distinguish these from varieties of *M. polymorpha* in O'ahu.

The patterns elucidated in our microsatellite study, taken in light of previous genetic and morphological studies, indicate that the genetic and morphological patterns of Hawaiian *Metrosideros* are likely due to a combination of chloroplast capture following hybridization, as well as morphological plasticity and parallel evolution on islands following rampant inter-island dispersal. The presence of genetic groups comprised of individuals with very different morphological characteristics may be the result of extreme morphological plasticity, where plants are able to change the characteristics of their vegetative parts due to fluctuations in environmental conditions [37], [38]. Previous studies have demonstrated that morphological characters of *M. polymorpha* change deterministically with environmental changes in temperature, precipitation, elevation, and substrate [6], [9], [10]. This plasticity and ecotypic differentiation may be important factors in the successful colonization of a wide and diverse landscape such as the Hawaiian Islands [37], [39].

The previous phylogenetic analysis of chloroplast data showed that *Metrosideros* in the Hawaiian Islands has island-based clades, and that diverse morphological forms are more closely related on any particular island than to similar forms on other islands [16]. The microsatellite analysis corroborates these general patterns but do not show such well-resolved island groups, with some individuals within each island sharing genetic similarity with individuals from other islands. This indicates that continued gene flow through inter-island dispersal is also likely occurring, which is consistent with the AMOVA results, where Fst values are more similar to plant groups with continental populations than isolated island populations. Such dispersal may occur by way of the tiny wind-dispersed seeds of *Metrosideros*, which only require wind speeds of 5–19 km per hour to be air-born [40], [41], or by the movement of pollen by birds. Two of the common native nectarivorous species of birds that visit *Metrosideros* show little or no evidence of genetic structure among islands [42], [43]. Inter-island movements of these birds could be a means of pollen exchange across islands.

Ancient chloroplast capture could not be ruled out as a partial explanation of the pattern of island-specific clades found in the chloroplast DNA study [16]. Chloroplast capture occurs when the usually maternally inherited chloroplast genome is transferred without recombination between species after hybridization [1]. The results of this nuclear microsatellite analysis therefore help to both corroborate the chloroplast findings and confirm the likelihood of chloroplast capture in some cases where there are different nuclear groups on an island, but only one chloroplast group. An example is *M. macropus*, which groups together but separate from the other taxa in O'ahu in the nuclear analysis, but in the chloroplast tree [16], these three individuals have identical chloroplast sequences to other taxa from O'ahu. Chloroplast capture has been identified in New Zealand *Metrosideros* [44] as well as other genera of Myrtaceae including *Eucalyptus* [45].

In addition to the microsatellite data revealing that the gene flow in Hawaiian *Metrosideros* is dynamic, results suggest that the current taxonomy is generally not valid and requires significant revisions. Results add mounting evidence that many *Metrosideros* taxa in the Hawaiian Islands should perhaps be regarded as one hypervariable species, *M. polymorpha*, including all varieties of *M. waialealae* and *M. polymorpha*, congruent with results of RAPD [14] and chloroplast DNA [16] studies. In addition, *M. polymorpha* vars. *dieteri* and *macrophylla* may be regarded as a single separate species, if any morphological characters can be identified to support this. Results from the structure analysis show that most individuals of *M. rugosa* and *M. tremuloides* form separate genetic groups, indicating that these two species may remain distinctive species, if further investigation reveals that taxonomic uncertainty, or misidentification, accounts for the few individuals that did not group with them (Figure 1*B*: red for *M. rugosa* and grey for *M. tremuloides*). Lastly, these results suggest that *M. collina* from the Austral Islands should be regarded as a separate species from *M. collina* from the Marquesas Islands, but combined with *M. macropus* from O'ahu.

The addition of the microsatellite data has broadened our interpretation of the biogeographic patterns in Hawaiian and French Polynesian *Metrosideros*, but some ambiguities still remain. Our data shows some conflict between admixture across islands and even across island groups in the Pacific, and a contrasting signature of intra-island lineages. If homoplasy in the microsatellite data is assumed to be low then the level of admixture implies an impressive degree of ongoing dispersal and gene flow between islands, which may account for Fst values that are more reflective of dispersal patterns seen in continental populations across a continuous land mass rather than the more typical Fst values reflective of restricted levels of dispersal between isolated islands interspersed by large areas of inhospitable ocean. We are currently pursuing a phylogenetic study of Hawaiian *Metrosideros* based on low-copy nuclear sequence data to resolve our interpretation of this system as well as the discrepancies between the chloroplast and microsatellite data.

## Supporting Information

Appendix S1 *Metrosideros* specimens used in this study. This list includes the following information: sample number (A: Figure 1A and Supporting Data; B: Figure 1B), voucher information, with herbarium abbreviations from the Index Herbariorum; the species; the variety if determined, or “indet.” if the variety is not determined; notes, on leaf surface characters (ie. glabrous or pubescent) of specimens for which the variety is not identified, or the specimen is intermediate in morphology.
(0.21 MB DOC)
