# Supplementary material for: Genetic Structure of the Polymorphic Metrosideros (Myrtaceae) Complex in the Hawaiian Islands Using Nuclear Microsatellite Data
Source: PLoS One. 2009 Mar 4;4(3):e4698. doi: 10.1371/journal.pone.0004698 (PMC2649539; doi:10.1371/journal.pone.0004698)
Supplement: Appendix S1 — Metrosideros specimens used in this study. This list includes the following information: sample number (A: Figure 1A and Supporting Data; B: Figure 1B), voucher information, with herbarium abbreviations from the Index Herbariorum; the species; the variety if determined, or “indet.” if the variety is not determined; notes, on leaf surface characters (ie. glabrous or pubescent) of specimens for which the variety is not identified, or the specimen is intermediate in morphology. (0.21 MB DOC) [file pone.0004698.s001.doc]

**Appendix S1. *Metrosideros* specimens used in this study.** This list includes the following information: sample number (A: Figure 1*A* and Supporting Data; B: Figure 1*B*), voucher information, with herbarium abbreviations from the Index Herbariorum; the species; the variety if determined, or “indet.” if the variety is not determined; notes, on leaf surface characters (ie. glabrous or pubescent) of specimens for which the variety is not identified, or the specimen is intermediate in morphology.[[1]](#endnote-2)

| **Sample# (A)** | **Sample**  **# (B)** | Voucher | **Species** | **Variety** | **Notes** | **Island** |
| --- | --- | --- | --- | --- | --- | --- |
| 1 | 1 | *Percy Tubuai1* (US) | *collina* |  |  | Austral Islands (Tubuai) |
| 2 | 2 | *Percy Tubuai2* (US) | *collina* |  |  | Austral Islands (Tubuai) |
| 3 | 3 | *Percy Tubuai3* (US) | *collina* |  |  | Austral Islands (Tubuai) |
| 4 | 4 | *Percy Tubuai4* (US) | *collina* |  |  | Austral Islands (Tubuai) |
| 5 | 5 | *Percy Rurutu50* (US) | *collina* |  |  | Austral Islands (Rurutu) |
| 6 | 6 | *Percy Tubuai9* (US) | *collina* |  |  | Austral Islands (Tubuai) |
| 7 | 7 | *Perlman 19743* (PTBG) | *collina* |  |  | Marquesas Islands (Ua Huka) |
| 8 | 8 | *Perlman 19672* (PTBG) | *collina* |  |  | Marquesas Islands (Fatu Hiva) |
| 9 | 9 | *Perlman 19733* (PTBG) | *collina* |  |  | Marquesas Islands (Ua Pou) |
| 10 | 10 | *Perlman 19734* (PTBG) | *collina* |  |  | Marquesas Islands (Ua Pou) |
| 11 | 11 | *Dunn 245* (PTBG) | *collina* |  |  | Marquesas Islands (Ua Pou) |
| 12 | 15 | *Wagner et al. 7010* (PTBG) | *polymorpha* | *dieteri* |  | Kaua`i |
| 13 | 16 | *Wagner et al.* *7012* (PTBG) | *polymorpha* | *dieteri* |  | Kaua`i |
| 14 | 17 | *Wagner et al. 7013* (PTBG) | *polymorpha* | *dieteri* |  | Kaua`i |
| 15 | 18 | *Wagner et al. 7041* (PTBG) | *polymorpha* | *dieteri* |  | Kaua`i |
| 16 | 19 | *Lorence et al. 9373* (PTBG) | *polymorpha* | *glaberrima* |  | Kaua`i |
| 17 | 25 | *Wagner et al. 7000* (PTBG) | *polymorpha* | *glaberrima* |  | Kaua`i |
| 18 | 26 | *Wagner et al. 7003* (PTBG) | *polymorpha* | *glaberrima* |  | Kaua`i |
| 19 | 27 | *Wagner et al.* *7004* (PTBG) | *polymorpha* | *glaberrima* |  | Kaua`i |
| 20 | 28 | *Wagner et al.* *7005* (PTBG) | *polymorpha* | *glaberrima* |  | Kaua`i |
| 21 | 29 | *Wagner et al. 7015* (PTBG) | *polymorpha* | *glaberrima* |  | Kaua`i |
| 22 | 30 | *Wagner et al. 7018* (PTBG) | *polymorpha* | *glaberrima* |  | Kaua`i |
| 23 | 31 | *Wagner et al. 7042* (PTBG) | *polymorpha* | *glaberrima* |  | Kaua`i |
| 24 | 32 | *Wagner et al. 7044* (PTBG) | *polymorpha* | *glaberrima* |  | Kaua`i |
| 25 | 33 | *Lorence 9370* (PTBG) | *polymorpha* | *glaberrima* |  | Kaua`i |
| 26 | 34 | *Lorence et al. 9379* (PTBG) | *polymorpha* | *glaberrima* |  | Kaua`i |
| 27 | 35 | *Lorence et al. 9371* (PTBG) | *polymorpha* | *glaberrima* |  | Kaua`i |
| 28 | 36 | *Lorence et al. 9372* (PTBG) | *polymorpha* | *glaberrima* |  | Kaua`i |
| 29 | 37 | *Wagner et al. 7007* (PTBG) | *polymorpha* | *glaberrima* |  | Kaua`i |
| 30 | 38 | *Wagner et al. 7017* PTBG | *polymorpha* | *glaberrima* |  | Kaua`i |
| 31 | 63 | *Wagner et al. 7008* (PTBG) | *polymorpha* | *incana* |  | Kaua`i |
| 32 | 92 | *Wagner et al. 7002* (PTBG) | *polymorpha* | indet. | intermediate | Kaua`i |
| 33 | 139 | Lorence et al. 9375  (PTBG) | *waialealae* | *waialealae* |  | Kaua`i |
| 34 | 140 | *Lorence et al. 9368* (PTBG) | *waialealae* | *waialealae* |  | Kaua`i |
| 35 | 141 | *Lorence et al. 9377* (PTBG) | *waialealae* | *waialealae* |  | Kaua`i |
| 36 | 142 | *Lorence et al. 9369* (PTBG) | *waialealae* | *waialealae* |  | Kaua`i |
| 37 | 143 | Morden 1360  (HAW) | *waialealae* | *waialealae* |  | Kaua`i |
| 38 | 12 | *Aradhya s.n.* | *macropus* |  |  | O`ahu |
| 39 | 13 | *Aradhya s.n.* | *macropus* |  |  | O`ahu |
| 40 | 14 | *Aradhya s.n.* | *macropus* |  |  | O`ahu |
| 41 | 108 | *Percy Hi54* (US) | *polymorpha* | *polymorpha** |  | O`ahu |
| 42 | 128 | *Morden s.n. †* | *rugosa* |  |  | O`ahu |
| 43 | 129 | *Morden s.n. †* | *rugosa* |  |  | O`ahu |
| 44 | 130 | *Morden s.n. †* | *rugosa* |  |  | O`ahu |
| 45 | 131 | *Wright 2.1* (COLO) | *rugosa* |  |  | O`ahu |
| 46 | 132 | *Wright 2.2 †* | *rugosa* |  |  | O`ahu |
| 47 | 109 | *Percy Hi49* (US) | *polymorpha* | *polymorpha* |  | O`ahu |
| 48 | 133 | *Morden s.n. †* | *tremuloides* |  |  | O`ahu |
| 49 | 134 | *Morden s.n. †* | *tremuloides* |  |  | O`ahu |
| 50 | 135 | *Morden s.n. †* | *tremuloides* |  |  | O`ahu |
| 51 | 136 | *Morden s.n. †* | *tremuloides* |  |  | O`ahu |
| 52 | 39 | *Wright 3.31* (COLO) | *polymorpha* | *glaberrima* |  | O`ahu |
| 53 | 40 | *Wright 3.32, †* | *polymorpha* | *glaberrima* |  | O`ahu |
| 54 | 41 | *Wright 5.33 †* | *polymorpha* | *glaberrima* |  | O`ahu |
| 55 | 42 | *Percy Hi4* (US) | *polymorpha* | *glaberrima* |  | O`ahu |
| 56 | 43 | Percy Hi10  (US) | *polymorpha* | *glaberrima* |  | O`ahu |
| 57 | 44 | *Percy Hi16* (US) | *polymorpha* | *glaberrima* |  | O`ahu |
| 58 | 110 | *Percy Hi9* (US) | *polymorpha* | *polymorpha* |  | O`ahu |
| 59 | 111 | Percy Hi12  (US) | *polymorpha* | *polymorpha* |  | O`ahu |
| 60 | 125 | *Wright 1.31* (COLO) | *polymorpha* | *pumila* |  | O`ahu |
| 61 | 126 | *Wright 1.32 †* | *polymorpha* | *pumila* |  | O`ahu |
| 62 | 127 | *Wright 1.33 †* | *polymorpha* | *pumila* |  | O`ahu |
| 63 | 45 | *Percy Hi51* (US) | *polymorpha* | *glaberrima* |  | O`ahu |
| 64 | 93 | *Percy Hi8* (US) | *polymorpha* | indet. | intermediate | O`ahu |
| 65 | 64 | *Percy Hi13* (US) | *polymorpha* | *incana** |  | O`ahu |
| 66 | 104 | *Morden s.n. †* | *polymorpha* | indet. | pubescent | O`ahu |
| 67 | 65 | *Percy Hi14* (US) | *polymorpha* | *incana* |  | O`ahu |
| 68 | 66 | *Percy Hi15* (US) | *polymorpha* | *incana* |  | O`ahu |
| 69 | 46 | *Percy Hi17* (US) | *polymorpha* | *glaberrima* |  | O`ahu |
| 70 | 137 | Percy *Hi18* (US) | *tremuloides* |  |  | O`ahu |
| 71 | 47 | *Percy Hi52* (US) | *polymorpha* | *glaberrima* |  | O`ahu |
| 72 | 48 | Percy Hi53  (US) | *polymorpha* | *glaberrima** |  | O`ahu |
| 73 | 20 | *Wood 11301* (PTBG) | *polymorpha* | *glaberrima* |  | Maui |
| 74 | 21 | *Price 290A* (US) | *polymorpha* | *glaberrima* |  | Maui |
| 75 | 22 | *Price 290B* (US) | *polymorpha* | *glaberrima* |  | Maui |
| 76 | 23 | *Price 290C* (US) | *polymorpha* | *glaberrima* |  | Maui |
| 77 | 105 | *Wright 02* (COLO) | *polymorpha* | *macrophylla* |  | Maui |
| 78 | 106 | Wright 03 † | *polymorpha* | *macrophylla* |  | Maui |
| 79 | 107 | *Wright 05 †* | *polymorpha* | *macrophylla* |  | Maui |
| 80 | 112 | *Price 290D* (US) | *polymorpha* | *polymorpha* |  | Maui |
| 81 | 113 | *Price 290E* (US) | *polymorpha* | *polymorpha* |  | Maui |
| 82 | 114 | *Price 290F* (US) | *polymorpha* | *polymorpha* |  | Maui |
| 83 | 124 | *Wright 01* (COLO) | *polymorpha* | *pseudorugosa* |  | Maui |
| 84 | 67 | *Percy Hi28* (US) | *polymorpha* | *incana** |  | Moloka`i |
| 85 | 49 | *Wood 11171* (PTBG) | *polymorpha* | *glaberrima* |  | Moloka`i |
| 86 | 50 | *Wood 11251* (PTBG) | *polymorpha* | *glaberrima* |  | Moloka`i |
| 87 | 51 | *Wood 11255* (PTBG) | *polymorpha* | *glaberrima* |  | Moloka`i |
| 88 | 52 | Percy Hi25  (US) | *polymorpha* | *glaberrima* |  | Moloka`i |
| 89 | 53 | Percy Hi26  (US) | *polymorpha* | *glaberrima* |  | Moloka`i |
| 90 | 54 | *Percy Hi30* (US) | *polymorpha* | *glaberrima* |  | Moloka`i |
| 91 | 68 | *Wood 11256* (PTBG) | *polymorpha* | *incana* |  | Moloka`i |
| 92 | 115 | *Wood 11250* (PTBG) | *polymorpha* | *polymorpha* |  | Moloka`i |
| 93 | 116 | *Wood 11259* (PTBG) | *polymorpha* | *polymorpha* |  | Moloka`i |
| 94 | 117 | Percy Hi24  (US) | *polymorpha* | *polymorpha* |  | Moloka`i |
| 95 | 69 | Percy Hi29  (US) | *polymorpha* | *incana* |  | Moloka`i |
| 96 | 138 | *Wood 11174* (PTBG) | *waialealae* | *fauriei* |  | Moloka`i |
| 97 | 24 | Percy Hi43  (US) | *polymorpha* | *glaberrima* |  | Hawai`i |
| 98 | 55 | Wright 5.31 (COLO) | *polymorpha* | *glaberrima* |  | Hawai`i |
| 99 | 56 | *Wright 5.32 †* | *polymorpha* | *glaberrima* |  | Hawai`i |
| 100 | 57 | *Price 275D* (US) | *polymorpha* | *glaberrima* |  | Hawai`i |
| 101 | 58 | *Price 275F* (US) | *polymorpha* | *glaberrima* |  | Hawai`i |
| 102 | 59 | *Wagner et al. 7035* (PTBG) | *polymorpha* | *glaberrima* |  | Hawai`i |
| 103 | 60 | *Wright 5.34 †* | *polymorpha* | *glaberrima* |  | Hawai`i |
| 104 | 61 | *Wood. 11155* (PTBG) | *polymorpha* | *glaberrima* |  | Hawai`i |
| 105 | 70 | *Wood et al. 11154* (PTBG) | *polymorpha* | *incana* |  | Hawai`i |
| 106 | 71 | Wagner et al. 7026  (PTBG) | *polymorpha* | *incana* |  | Hawai`i |
| 107 | 72 | *Wagner et al. 7028* (PTBG) | *polymorpha* | *incana* |  | Hawai`i |
| 108 | 73 | *Wagner et al. 7029* (PTBG) | *polymorpha* | *incana* |  | Hawai`i |
| 109 | 74 | *Wagner et al. 7030* (PTBG) | *polymorpha* | *incana* |  | Hawai`i |
| 110 | 75 | *Wagner et al. 7032* (PTBG) | *polymorpha* | *incana* |  | Hawai`i |
| 111 | 76 | *Wagner et al. 7033* (PTBG) | *polymorpha* | *incana* |  | Hawai`i |
| 112 | 77 | *Wagner et. al. 7036* (PTBG) | *polymorpha* | *incana** |  | Hawai`i |
| 113 | 118 | *Price 275A* (US) | *polymorpha* | *polymorpha* |  | Hawai`i |
| 114 | 119 | *Price 275B* (US) | *polymorpha* | *polymorpha* |  | Hawai`i |
| 115 | 120 | *Price 275C* (US) | *polymorpha* | *polymorpha* |  | Hawai`i |
| 116 | 78 | *Wagner et al. 7031* (PTBG) | *polymorpha* | *incana* |  | Hawai`i |
| 117 | 121 | *Wright 4.31* (COLO) | *polymorpha* | *polymorpha** |  | Hawai`i |
| 118 | 122 | *Wright 4.32 †* | *polymorpha* | *polymorpha** |  | Hawai`i |
| 119 | 123 | *Wright 4.33 †* | *polymorpha* | *polymorpha** |  | Hawai`i |
| 120 | 85 | *Morden s.n. †* | *polymorpha* | indet. | glabrous | Hawai`i |
| 121 | 86 | *Morden s.n. †* | *polymorpha* | indet. | glabrous | Hawai`i |
| 122 | 87 | *Morden s.n. †* | *polymorpha* | indet. | glabrous | Hawai`i |
| 123 | 88 | *Morden s.n. †* | *polymorpha* | indet. | glabrous | Hawai`i |
| 124 | 89 | *Morden s.n. †* | *polymorpha* | indet. | glabrous | Hawai`i |
| 125 | 90 | *Morden s.n. †* | *polymorpha* | indet. | glabrous | Hawai`i |
| 126 | 62 | *Percy Hi36* (US) | *polymorpha* | *glaberrima* |  | Hawai`i |
| 127 | 91 | *Percy Hi44*  (US) | *polymorpha* | indet. | glabrous | Hawai`i |
| 128 | 94 | *Percy Hi41*  (US) | *polymorpha* | indet. | intermediate | Hawai`i |
| 129 | 95 | *Morden s.n. †* | *polymorpha* | indet. | intermediate | Hawai`i |
| 130 | 96 | *Morden s.n. †* | *polymorpha* | indet. | intermediate | Hawai`i |
| 131 | 97 | *Morden s.n. †* | *polymorpha* | indet. | intermediate | Hawai`i |
| 132 | 79 | *Wagner et al. 7041* (PTBG) | *polymorpha* | *incana* |  | Hawai`i |
| 133 | 98 | *Morden s.n. †* | *polymorpha* | indet. | pubescent | Hawai`i |
| 134 | 99 | *Morden s.n. †* | *polymorpha* | indet. | pubescent | Hawai`i |
| 135 | 100 | *Morden s.n. †* | *polymorpha* | indet. | pubescent | Hawai`i |
| 136 | 101 | *Morden s.n. †* | *polymorpha* | indet. | pubescent | Hawai`i |
| 137 | 102 | *Morden s.n. †* | *polymorpha* | indet. | pubescent | Hawai`i |
| 138 | 103 | *Morden s.n. †* | *polymorpha* | indet. | pubescent | Hawai`i |
| 139 | 80 | *Percy Hi34*  (US) | *polymorpha* | *incana* |  | Hawai`i |
| 140 | 81 | *Percy Hi47*  (US) | *polymorpha* | *incana* |  | Hawai`i |
| 141 | 82 | *Percy Hi31*  (US) | *polymorpha* | *incana* |  | Hawai`i |
| 142 | 83 | *Percy Hi35*  (US) | *polymorpha* | *incana* |  | Hawai`i |
| 143 | 84 | *Percy Hi42* (US) | *polymorpha* | *incana* |  | Hawai`i |

1. *†* Specimens for which an herbarium voucher could not be located.

   * Taxa with updated identifications from chloroplast DNA phylogeny study [16]. [↑](#endnote-ref-2)
